# Inhibition of calpain reduces oxidative stress and attenuates endothelial dysfunction in diabetes

**DOI:** 10.1186/1475-2840-13-88

**Published:** 2014-05-03

**Authors:** Bainian Chen, Qing Zhao, Rui Ni, Futian Tang, Limei Shan, Inga Cepinskas, Gediminas Cepinskas, Wang Wang, Peter W Schiller, Tianqing Peng

**Affiliations:** 1 Institute for Cardiovascular Science of Soochow University, Suzhou, Jiangsu Province 215123, China; 2 Lawson Health Research Institute, London Health Sciences Centre, London N6A 4G5, Ontario, Canada; 3 Department of Medicine, University of Western Ontario, London N6A 4G5, Ontario, Canada; 4 Department of Pathology, University of Western Ontario, London N6A 4G5, Ontario, Canada; 5 Department of Cardiology, Shanghai 6th People’s Hospital, Shanghai Jiaotong University School of Medicine, Shanghai 200233, China; 6 Mitochondria and Metabolism Center, Departments of Anesthesiology and Pain Medicine, University of Washington, Seattle, USA; 7 Laboratory of Chemical Biology and Peptide Research, Clinical Research Institute of Montreal, Montreal, Quebec H2W 1R7, Canada; 8 Critical Illness Research, Lawson Health Research Institute, VRL 6th Floor, A6-140, 800 Commissioners Road, London, Ontario, Canada

**Keywords:** Diabetes, Calpain, eNOS, ROS, Endothelial dysfunction

## Abstract

**Aims:**

The present study was to investigate the role of calpain in reactive oxygen species (ROS) production in endothelial cells and endothelium-dependent vascular dysfunction under experimental conditions of diabetes.

**Methods and results:**

Exposure to high glucose activated calpain, induced apoptosis and reduced nitric oxide (NO) production without changing eNOS protein expression, its phosphorylation and dimers formation in primary human umbilical vein endothelial cells (HUVECs). These effects of high glucose correlated with intracellular ROS production and mitochondrial superoxide generation. Selectively scavenging mitochondrial superoxide increased NO production in high glucose-stimulated HUVECs. Inhibition of calpain using over-expression of calpastatin or pharmacological calpain inhibitor prevented high glucose-induced ROS production, mitochondrial superoxide generation and apoptosis, which were concurrent with an elevation of NO production in HUVECs. In mouse models of streptozotocin-induced type-1 diabetes and OVE26 type-1 diabetic mice, calpain activation correlated with an increase in ROS production and peroxynitrite formation in aortas. Transgenic over-expression of calpastatin reduced ROS production and peroxynitrite formation in diabetic mice. In parallel, diabetes-induced reduction of endothelium-dependent relaxation in aortic ring was reversed by over-expression of calpastatin in mouse models of diabetes. However, the protective effect of calpastatin on endothelium-dependent relaxation was abrogated by eNOS deletion in diabetic mice.

**Conclusions:**

This study suggests that calpain may play a role in vascular endothelial cell ROS production and endothelium-dependent dysfunction in diabetes. Thus, calpain may be an important therapeutic target to overcome diabetes-induced vascular dysfunction.

## Introduction

Diabetes mellitus is one of the worldwide leading causes of death and long-term disability, resulting in huge social and economic burden. Vascular abnormalities are the major contributor to the progression of diabetes and its associated complications [1]. Because the endothelium is an important component of vascular homeostasis and the primary target of hyperglycemia and hyperlipidemia, endothelial dysfunction occurs in both animal models of diabetes and diabetic patients [2-5] and it has been implicated in diabetic vascular complications [1,2]. Although the pathophysiology of diabetic endothelial dysfunction is incompletely characterized, it appears to be multifactorial. Amongst various proposed mechanisms, oxidative damage induced by reactive oxygen species (ROS) has been critical in this disorder [6]. Exposure of endothelial cells to high glucose induces ROS production *in vitro*[7,8]. Clinical and animal studies also demonstrate an increase in vascular ROS formation in diabetes [9,10]. Importantly, treatment with antioxidants improves endothelial-dependent vascular relaxation in animal models of diabetes [11], supporting a central role of ROS in the development of diabetic endothelial dysfunction. Multiple mechanisms have been proposed to be responsible for ROS production in diabetes. In addition to enhanced glucose auto-oxidation, increased substrate flux through the polyol pathway and stimulation of eicosanoid metabolism, ROS is mainly produced by mitochondria, NADPH oxidase, xanthine oxidase and un-coupled nitric oxide (NO) synthase (NOS) [12-14]. Both xanthine oxidase and NADPH oxidase have been reported to induce ROS production in diabetic vessels, which significantly contributes to endothelial dysfunction [15-17]. Hyperglycemia-induced mitochondrial respiratory chain deficiencies are postulated to be another critical and unifying source of ROS generation [15]. Mitochondria-derived superoxide production may be the initiator for a vicious cycle of oxidative stress in diabetes [18]. Excessive ROS production also induces a dysfunctional eNOS, or referred as eNOS uncoupling, which generates superoxide instead of NO [3,15]. Increased production of superoxide in endothelial cells reacts directly with NO to form a more harmful molecule peroxynitrite (ONOOˉ), thereby reducing NO bioavailability [2,3]. Elevated ROS production and reduced NO bioavailability, together with the intermediate product peroxynitrite, significantly account for apoptosis in endothelial cells and endothelial dysfunction in diabetes [19,20]. However, the regulation of ROS generation has not been fully addressed in diabetes.

Calpains belong to a family of calcium-dependent thiol-proteases. They have been involved in a wide variety of cellular processes including remodeling of cytoskeletal, caspases activation/apoptosis and acute inflammation [21]. Two major isoforms of calpain, calpain-1 and calpain-2, are ubiquitously expressed, and calpastatin is an endogenous inhibitor for calpain-1 and calpain-2 [21]. Over-expression of calpastatin is shown to inhibit calpain activity *in vitro* and in transgenic mice [22]. Calpain activity is increased in endothelial cells under diabetic conditions [23,24]. An early study showed that inhibition of calpain increased NO production from eNOS and reduced leukocyte-endothelium interactions in microcirculation during hyperglycemia [25]. These effects of calpain inhibition were further confirmed in a genetic rat model of type 2 diabetes [24,26]. It has been also suggested that calpain activation contributes to microvascular albumin leakage in diabetes [26]. Nevertheless, the role of calpain in diabetic vascular complications has not been fully characterized. Particularly, the functional significance of calpain remains to be determined and whether calpain plays a role in regulating ROS production has never been reported in diabetic endothelial dysfunction.

In the present study, we employed an *in vitro* model of endothelial cells stimulated with high glucose and multiple *in vivo* models of diabetes to investigate the role of calpain in ROS generation and endothelial dysfunction.

## Methods

### Animals

This investigation conforms to the Guide for the Care and Use of Laboratory Animals published by the US National Institutes of Health (NIH Publication, 8^th^ Edition, 2011). All experimental procedures were approved by the Animal Use Subcommittee at the University of Western Ontario,Canada. Breeding pairs of C57BL/6 mice, FVB(Cg)-Tg(Ins2-CALM)26OveTg(Cryaa-Tag)1Ove/PneJ transgenic mice (OVE26, a mouse model of type 1 diabetes), db/db mice (a mouse model of type 2 diabetes), and eNOS knockout mice (eNOS-KO) were purchased from the Jackson Laboratory. Transgenic mice over-expressing calpastatin (Tg-CAST) driven by cytomegalovirus promoter were provided by Dr. Laurent Baud (the Institut National de la Santé et de la Recherche Médicale, Paris, France) through the European Mouse Mutant Archive [27]. OVE26/Tg-CAST, eNOS-KO/Tg-CAST and db/db/Tg-CAST mice were generated by breeding Tg-CAST with OVE26, eNOS-KO and db/+/− mice, respectively. Type 1 diabetes was induced in adult male mice (10–15 mice in each group) by intrapenitoneally (i.p.) injection with streptozotocin (STZ, 50 mg/kg/day) for 5 consecutive days as described in our recent reports [28]. The mice were considered diabetic and used for the study only if they had hyperglycemia (>15 mmol/L) at 72 h after the last injection of STZ, whereas citrate buffer-treated mice were used as non-diabetic control (blood glucose < 12 mmol/L). Two months after STZ injection, animals were killed by cervical dislocation with anesthesia (ketamine: 100 mg/kg, i.p.) and tissues collected for the following analyses.

### Cell culture and adenoviral infection

Human umbilical vein endothelial cells (HUVECs) were isolated from umbilical cord veins and cultured as we previously described [29]. The isolation of the HUVECs was performed conforming the declaration of Helsinki and approved by the ethics review board at the University of Western Ontario. Cells at passage 1–5 were used in this study.

HUVECs were infected with adenoviral vectors containing rat calpastatin gene (Ad-CAST, University of Buffalo, USA) or beta-gal (Ad-gal, Vector Biolabs) as a control at a multiplicity of infection (MOI) of 10 PFU/cell. Adenovirus-mediated gene transfer was implemented as previously described [30]. All experiments were performed after 24 hours of adenoviral infection.

### Drugs

D-glucose, STZ, phenylephrine (PE), acetylcholine (Ach), sodium nitroprusside (SNP), mito-TEMPO and calpain inhibitor-III were purchased from Sigma or Calbiochem. Hoechst 33324, 2,7- dichlorofluorescein diacetate (DCF-DA) and dihydroethidium (DHE) were from Invitrogen. The mitochondria-targeted antioxidant peptide SS31 and peptide SS20 which lacks antioxidant properties were synthesized as described previously [31].

### Calpain activity

Calpain activity was determined in cell or tissue lysates by using a fluorescence substrate *N*-succinyl-LLVY-AMC (Cedarlane Laboratories) as described previously [28].

### ROS production

Intracellular ROS production was detected by using DCF-DA. Briefly, HUVECs were washed with phosphate-buffered saline and loaded with freshly prepared DCF-DA (10 μmol/L) for 15 minutes at 37°C. Hoechst 33324 was used to stain nucleus. The DCF-DA signals were recorded by a fluorescence microscope. Aortic tissues were homogenized and ROS production was measured in tissue lysates using DCF-DA and DHE probes as described in our recent report [30].

### Single mitochondrial superoxide generation

Superoxide flashes in single mitochondrion were measured to determine mitochondrial superoxide generation in living endothelial cells as described previously [32]. Briefly, HUVECs were infected with an adenoviral vector expressing mt-cpYFP (Ad-mt-cpYFP). Ad-mt-cpYFP expresses a circularly permuted yellow fluorescent protein (cpYFP) in the mitochondrial matrix of cells using the cytochrome C oxidase subunit IV targeting sequence (mt-cpYFP). Twenty-four hours after infection, confocal imaging was recorded using the Olympus FV 1000 laser-scanning microscope equipped with a 63x, 1.3NA oil immersion objective and a sampling rate of 0.7 s/frame. The mitochondria-targeted superoxide dismutase analogue mito-TEMPO was used to confirm superoxide generation during measurements.

### Nitric oxide production

The cell culture media were collected and assayed for NO production using a commercial kit (Cayman Chemical Company) according to the manufacturer’s instructions. Briefly, cell culture medium was harvested and centrifuged at 1000 g for 2 minutes. Twenty μL of the supernatant of each sample was incubated with nitrate reductase mixture for 1 hour at room temperature. The NO level was examined through detection of the fluorescent product 1(H)-naphthotriazole formed from the reaction between 2,3-diaminonaphthalene and nitrite at an excitation wavelength of 360 nm and an emission wavelength of 430 nm.

### Caspase-3 activity

Caspase-3 activity in HUVECs was measured using a fluorescent assay kit (BIOMOL Research Laboratories) as described in our previous reports [31].

### Annexin V staining

HUVECs were incubated with annexin V-conjugated with FITC and Hoechst 33324 in culture medium as described previously [33]. At least 200 cells were examined from each sample.

### Western blot analysis

The protein levels of phosphorylated eNOS (S1179), eNOS, eNOS dimers and GAPDH were determined by western blot analysis using specific antibodies against the corresponding proteins (Cell Signaling), respectively.

### Peroxynitrite

The formation of peroxynitrite in the aortas of diabetic mice was assessed by the immunofluorescent staining with a primary antibody against nitrotyrosine (Cayman Chemical Company, dilution 1:100) according to a standard immunohistofluorescent staining procedure as described previously [34].

### Relaxation of aortic ring

Mouse aortic ring was prepared and precontracted with phenylephrine, and endothelium dependent relaxation was produced by the addition of acetylcholine as described previously [35,36]. The vaso-relaxations were expressed as percentage dilation of PE-induced pre-constriction.

### Statistical analysis

All data were given as mean ± SD. Differences between two groups were compared by unpaired Student’s t-test. For multi-group comparisons, ANOVA followed by Newman-Keuls test was performed. A two-tailed value of *P* < 0.05 was regarded as statistically significant.

## Results

### Over-expression of calpastatin reduces ROS production in endothelial cells

**Figure 1:**
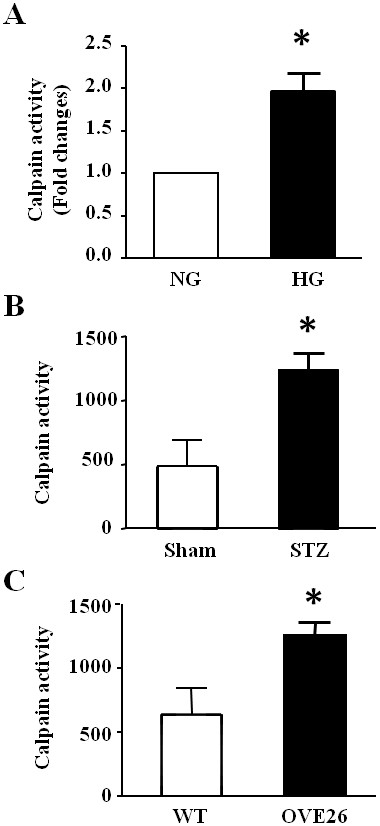
**Effect of high glucose or diabetes on calpain activity. (A)** Calpain activity in HUVECs incubated with normal glucose (NG, 5 mmol/L) or high glucose (HG, 30 mmol/L) for 48 h. Data are given as mean ± SD from 3 different experiments. **(B)** Calpain activity in the aortas of sham and STZ-induced diabetic mice. **(C)** Calpain activity in the aortas of wild-type (WT) and transgenic type 1 diabetic OVE26 mice. Data are given as mean ± SD, n = 6. ^*^P < 0.05 vs. NG group, sham mice or WT mice.

**Figure 2:**
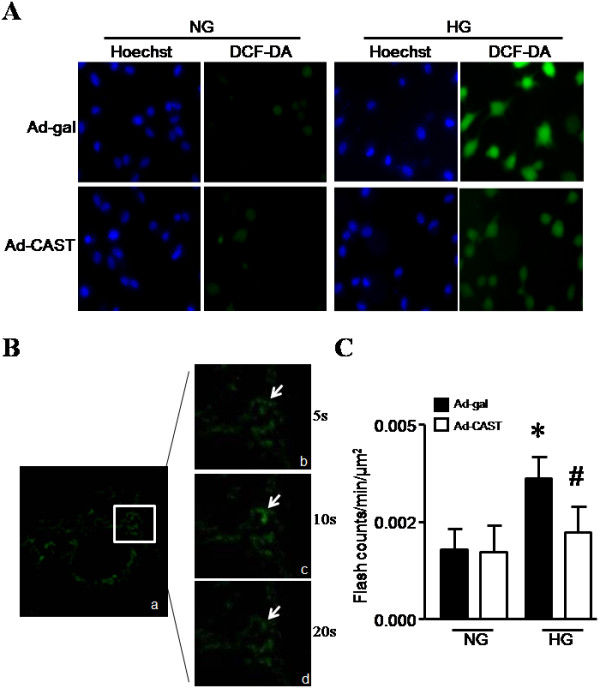
**Measurement of ROS production in HUVECs.** HUVECs were incubated with normal glucose (NG, 5 mmol/L) or high glucose (HG, 30 mmol/L) in combination with infection with Ad-gal (adenoviral vectors containing beta-gal as a control) or Ad-CAST for 48 h. **(A)** ROS formation in HUVECs was determined by DCF-DA staining and the nucleus was identified by Hoechst33342 staining. Representative fluorescent pictures of Hoechst33342 (blue signal) and DCF-DA staining (green signal) are shown from 3 different experiments. **(B, C)** Mitochondrial superoxide flashes were measured in HUVECs. Representative confocal images for the time-course of superoxide flash occurrence from at least 5 cells per experiment in each group. Arrow indicates superoxide flash in a single mitochondrion **(B)**. Quantification of superoxide flashes per μm^2^ in every 60 seconds **(C)**. Data are given as mean ± SD from 4 different experiments. ^*^P < 0.05 vs. NG treated with vehicle or infected with Ad-gal. ^#^*P* < 0.05 vs. HG treated with vehicle or infected with Ad-gal.

In agreement with a previous report [24], we showed that calpain activity was increased in HUVECs by high glucose compared with normal glucose (*P* < 0.05, Figure 1A). High glucose also significantly induced ROS production in HUVECs as shown by enhanced DCF-DA fluorescence (Figure 2A). To investigate the role of calpain in ROS production, we over-expressed calpastatin in HUVECs by infection with Ad-CAST and then incubated these cells with normal (5 mmol/L) or high glucose (30 mmol/L) for 48 hours. Ad-gal was used as a control. Infection of HUVECS with Ad-CAST significantly decreased calpain activity (Additional file 1: Figure S1 Effect of calpastatin over-expression on calpain activity) and the density of DCF-DA staining following high glucose-stimulation, indicating that inhibition of calpain prevents ROS production (Figure 2A).

Since mitochondrion is an important source of ROS production under physiological and pathological conditions [37], we investigated whether calpain plays a role in mitochondrial ROS generation. To this end, we measured single mitochondrion superoxide generation in living endothelial cells. HUVECs were infected with Ad-mpYFP in combination with Ad-CAST or Ad-gal and then incubated with normal glucose or high glucose for 24 hours. The mitochondrial superoxide flashes were recorded using confocal microscope (Figure 2B). High glucose significantly increased the frequency of mitochondrial superoxide flashes in HUVECs as compared with normal glucose (0.0036 *vs* 0.0018, *P* < 0.05). In contrast, high glucose-induced mitochondrial superoxide flashes were reduced by over-expression of calpastatin (Figure 2C). These data suggest that calpain activation contributes to mitochondrial ROS generation in endothelial cells during high glucose stimulation.

### Inhibition of calpain increases NO production in endothelial cells by reducing ROS generation

**Figure 3:**
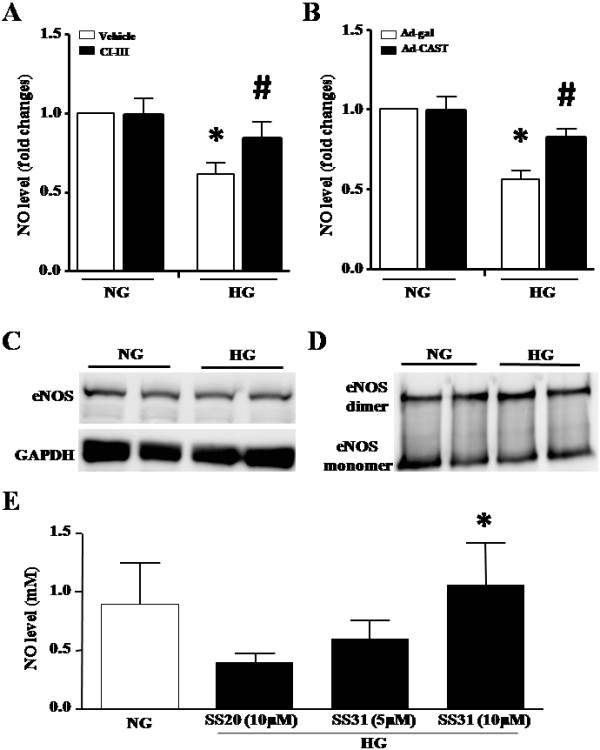
**Measurement of nitric oxide production and eNOS protein in HUVECs. (A, B)** Effects of calpain inhibition on nitric oxide production. HUVECs were incubated with normal glucose (NG, 5 mmol/L) or high glucose (HG, 30 mmol/L) in combination with calpain inhibitor-III (CI-III, 10 μmol/L) or infection with Ad-CAST for 48 h. NO production was measured in culture medium. Data are given as mean ± SD from at least 3 different experiments. ^*^P < 0.05 vs. NG treated with vehicle or infected with Ad-gal. ^#^*P* < 0.05 vs. HG treated with vehicle or infected with Ad-gal. **(C, D)** Effect of high glucose on eNOS. HUVECs were incubated with NG or HG for 48 h. The protein levels of eNOS **(C)**, and eNOS monomer and dimmer were determined **(D)**. **(C, D)** are representative western blots for eNOS and dimmer formation from 3 different experiments. **(E)** Nitric oxide (NO) productions. HUVECs were incubated with high glucose in the presence of mitochondria-targeted antioxidant peptide SS31 (5 or 10 μmol/L) or control peptide SS20 (10 μmol/L) for 48 h. NO production was measured in culture medium. Data are given as mean ± SD from 3 different experiments. ^*^P < 0.05 vs. SS20.

NO production was dramatically decreased in HUVECs in response to high glucose (*P* < 0.05, Figure 3A and B). However, the levels of eNOS protein and phosphorylated eNOS (Additional file 1: Figure S2 Effects of calpain inhibition on phosphorylated eNOS in HUVECs), and the formation of eNOS dimers were not changed during high glucose incubation (Figure 3C and D). This suggests that the function of eNOS may be not impaired by high glucose and thus, a reduction in NO production may be not due to the abnormality of eNOS in high glucose-stimulated HUVECs. It has been well-known that NO can be quenched by ROS. Interaction of NO with superoxide anion leads to the formation of peroxynitrite [1,3]. Indeed, inhibition of mitochondrial ROS with a mitochondrial-targeted antioxidant SS31 increased NO production in high glucose-stimulated HUVECs in a dose-dependent manner (Figure 3E). This result argues that ROS produced by mitochondria accounts for the reduction in NO production in endothelial cells during high glucose stimulation.

Having shown that calpain is important in producing ROS, we hypothesized that calpain activation reduced NO production in high glucose-induced HUVECs. In support of our hypothesis, pharmacological inhibition of calpain with calpain inhibitor-III or over-expression of calpastatin significantly increased NO levels in high glucose-stimulated HUVECs (*P* < 0.05, Figure 3A and B). However, the protein levels of phosphorylated eNOS were not changed (Additional file 1: Figure S2 Effects of calpain inhibition on phosphorylated eNOS in HUVECs).

### Apoptosis is attenuated by calpain inhibition

**Figure 4:**
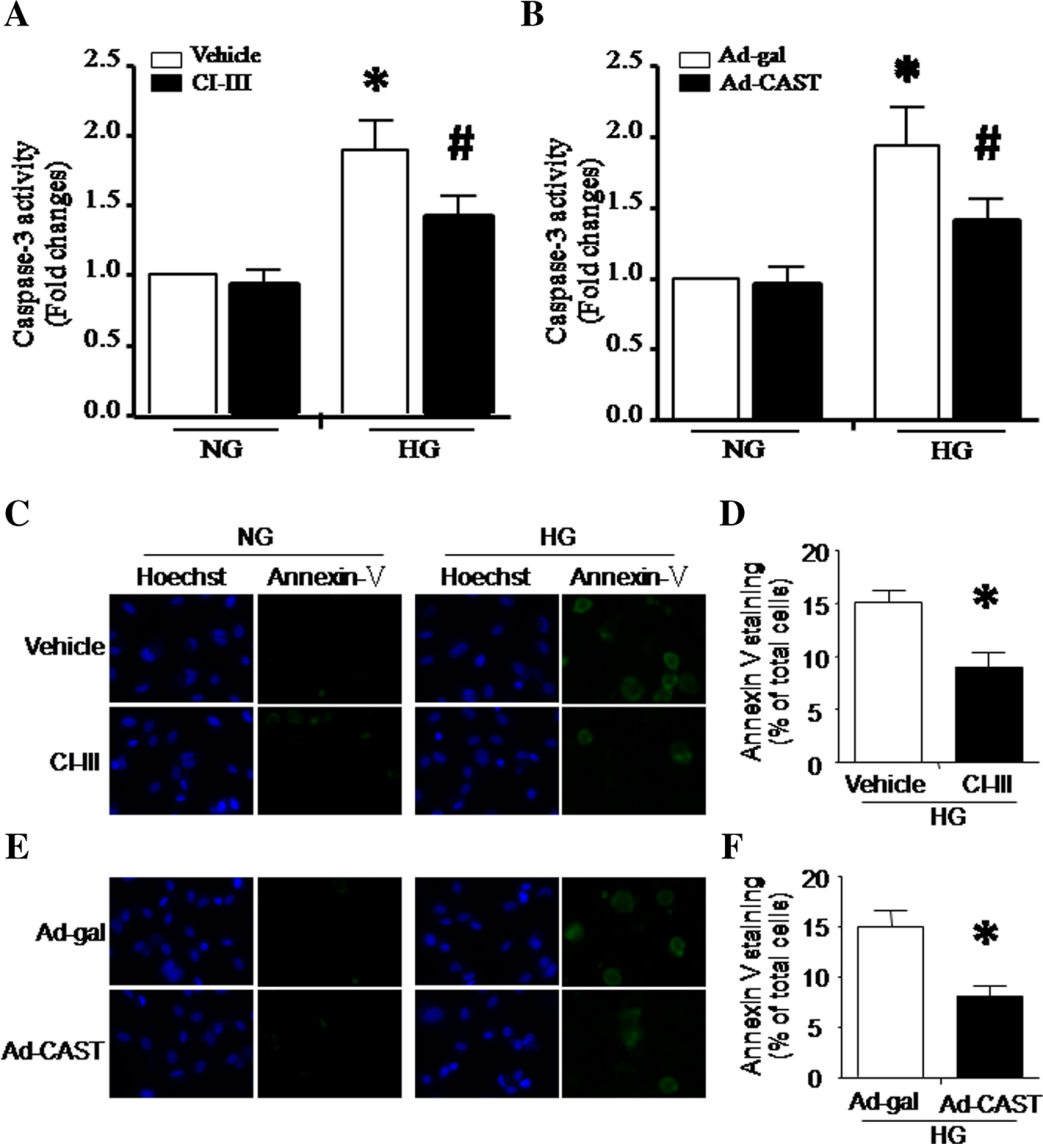
**Effect of calpain inhibition on apoptosis in high glucose-stimulated HUVECs.** HUVECs were incubated with normal glucose (NG, 5 mmol/L) or high glucose (HG, 30 mmol/L) in combination with calpain inhibitor-III (CI-III, 10 μmol/L) or infection with Ad-CAST for 48 h. **(A, B)** Caspase-3 activity. **(C, E)** Representative images for annexin V staining in HUVECs (Green). **(D, F)** Quantification of annexin V staining positive cells. Data are given as mean ± SD from 3 different experiments. ^*^P < 0.05 vs. NG treated with vehicle or infected with Ad-gal. ^#^*P* < 0.05 vs. HG treated with vehicle or infected with Ad-gal.

To investigate whether inhibition of calpain would prevent apoptosis induced by high glucose, we treated HUVECs with normal glucose or high glucose in the presence of calpain inhibitor-III (10 μmol/L) or vehicle for 48 hours. Consistent with previous reports [8,20], high glucose resulted in apoptosis in HUVECs as determined by caspase-3 activity and annexin V staining (Figure 4). Calpain inhibitor-III remarkably decreased apoptosis in high glucose-stimulated HUVECs (Figure 4A, C and D). Similarly, over-expression of calpastatin significantly inhibited apoptosis induced by high glucose (Figure 4B, E and F). These data demonstrate that inhibition of calpain attenuates high glucose-induced apoptosis in endothelial cells.

### Over-expression of calpastatin reduces ROS production and peroxynitrite in aortic tissues from diabetic mice

**Figure 5:**
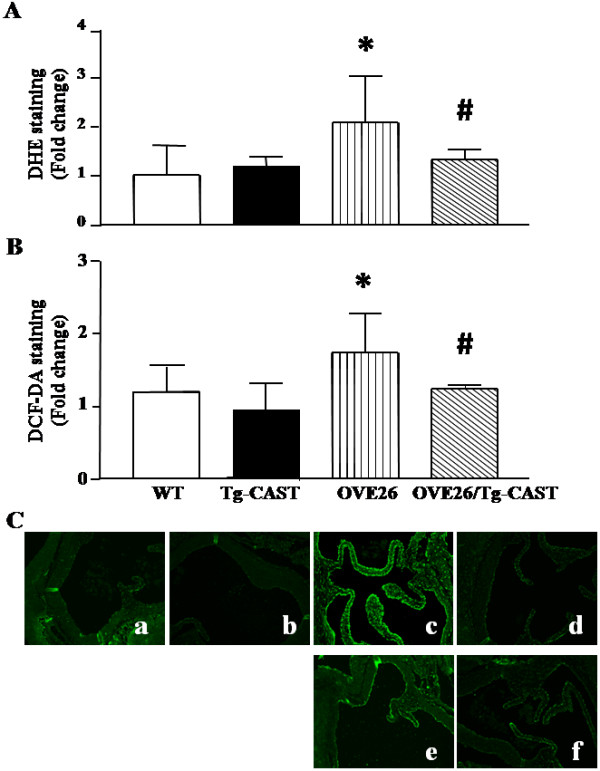
**Effect of calpain inhibition on the ROS production and peroxynitrite formation in the aortas of diabetic mice. (A, B)** ROS formation in the vessels assessed by DHE and DCF-DA staining, respectively. **(C)** Representative images for peroxynitrite formation in aortas from 4 mice in each group. a, WT mice; b, Tg-CAST mice; c, OVE26 mice; d, OVE26/Tg-CAST mice; e, STZ-injected wild-type (WT) mice; f, STZ-injected Tg-CAST mice; Data are given as mean ± SD, n = 6. ^*^P < 0.05 vs. WT mice. ^#^*P* < 0.05 vs. OVE26 mice.

Calpain activity in aortas of STZ-induced diabetic mice and type-1 diabetic OVE26 mice was increased about 2 folds compared to the non-diabetic mice (*P* < 0.05, Figure 1B and C). To determine whether calpain contributes to ROS production in vascular tissues from diabetic mice *in vivo*, ROS formation was measured in aortas of wild-type, OVE26 and double transgenic OVE26/Tg-CAST mice. Compared to wild-type mice, ROS production in diabetic OVE26 mouse aortas was significantly increased. Over-expression of calpastatin significantly decreased ROS production in the vessels of OVE26/Tg-CAST mice. These results suggest that calpain activation plays a role in vascular ROS production *in vivo* (Figure 5A and B).

Since superoxide anion is able to interact with NO to form peroxynitrite [2], we determined the formation of peroxynitrite in diabetic mouse aortas. As shown in Figure 5C, STZ-induced and OVE26 mice displayed a significant formation of peroxynitrite in aortic endothelium compared to the vessels of non-diabetic controls, confirming an activated status of nitrated tyrosine in diabetic vessels [9]. Over-expression of calpastatin inhibited the vascular peroxynitrite formation, supporting an important role of calpain activity in nitro-oxidative stress in diabetic vasculature.

### Over-expression of calpastatin improves nitric oxide-dependent vascular relaxation in diabetic mice

**Figure 6:**
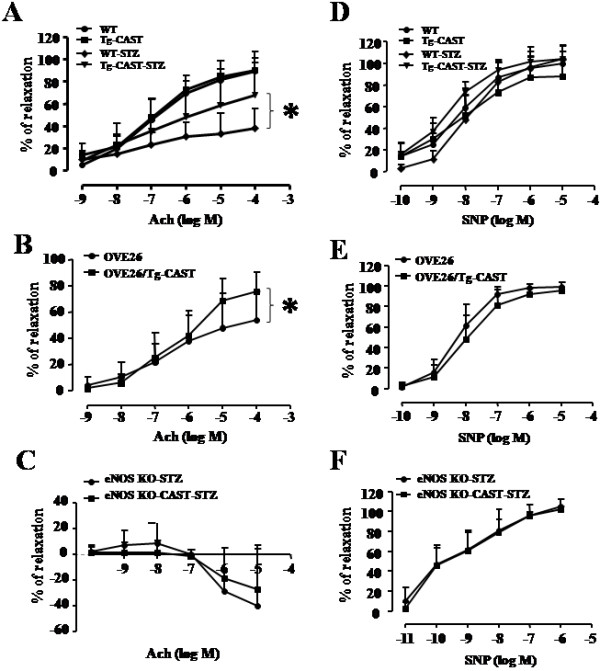
**Role of calpain in endothelium-dependent and -independent relaxation.** Aortic rings were pre-contracted by phenylephrine and the subsequent responses to acetylcholine (Ach) or sodium nitroprusside (SNP) were recorded. **(A-C)** the response to Ach in STZ-induced mice **(A)**, OVE26 mice **(B)**, and eNOS knockout mice **(C) (D-F)**. the response to SNP in STZ-induced mice (D), OVE26 mice **(E)**, and eNOS knockout mice **(F)**. Data are given as mean ± SD, n = 6-8. *P < 0.05.

Endothelium-dependent vaso-relaxation represents a fundamental parameter for the function of endothelium in vessels. Impaired endothelium-dependent relaxation is observed in both diabetic patients and animal models [9-11]. Consistently, our results showed that the relaxation in response to Ach was markedly blunted by more than 50% in aortic rings of STZ-induced diabetic mice compared with sham animals (*P* < 0.05, Figure 6A). In contrast, the vasodilatory function of vascular smooth muscle assessed by the response to SNP remained comparable between diabetic and non-diabetic aortic rings (Figure 6D). These results indicate that endothelium-dependent relaxation but not endothelium-independent vascular function is compromised in our models of diabetes, suggesting that the bioavailability of endothelium-derived NO is reduced in diabetes. However, transgenic over-expression calpastatin significantly improved the vascular relaxation in STZ-induced Type-1 diabetic mice (*P* < 0.05, Figure 6A). To further address the role of calpain in endothelial dysfunction, we evaluated endothelium-dependent relaxation in aortic rings from OVE26 mice. Consistently, the cumulative concentration-response of aortic rings to Ach was significantly decreased in OVE26 mice compared with their non-diabetic littermates. Transgenic over-expression of calpastatin restored the vascular relaxation back to the normal levels (Figure 6B). Similarly, the protective effect of calpastatin over-expression on endothelial function was also observed in a mouse model of type-2 diabetes, db/db mice (data not shown). Taken together, over-expression of calpastatin reduces endothelium-dependent dysfunction in diabetes.

### Deletion of eNOS abrogates the effect of calpastatin on endothelium-dependent relaxation in diabetic mice

To investigate whether the functional improvement by calpain inhibition was mediated through eNOS-derived NO pathway, we induced diabetes in eNOS-KO and eNOS-KO/Tg-CAST mice by STZ injection. Two months after STZ injection, the blood glucose levels and blood pressure were comparable between eNOS-KO and eNOS-KO/Tg-CAST mice (data not shown). We then evaluated the vascular function by measuring endothelium-dependent relaxation. Our results showed that the response of aortic rings to Ach was almost abolished in eNOS-KO mice while the response to SNP remained unchanged (Figure 6C and F). Over-expression of calpastatin did not improve this response in diabetic eNOS-KO/Tg-CAST mice. These findings further confirm that the eNOS/NO pathway but not other vasodilatory factors are involved in calpain-mediated endothelial dysfunction during diabetes.

## Discussion

The current study investigated the role of calpain in endothelial ROS production and endothelial dysfunction during diabetes. Herein, we demonstrate for the first time that inhibition of calpain reduces mitochondrial superoxide generation, intracellular ROS production and apoptotic cell death in high glucose-stimulated endothelial cells. The effects of calpain inhibition correlate with an elevation of NO production and selectively scavenging mitochondrial ROS increases NO production during high glucose stimulation. In mouse models of type-1 and type-2 diabetes, transgenic over-expression of calpastatin reduces vascular ROS production, inhibits peroxynitrite formation, and attenuates the dysfunction of endothelium-dependent relaxation. Thus, this study reveals a novel role of calpain activation in endothelial ROS generation under diabetic conditions. Excessive ROS possibly produced by mitochondria quenches NO, thereby generating toxic oxidant species peroxynitrite and reducing NO bio-availability and thus, induces endothelial dysfunction in diabetes.

Studies have demonstrated that high glucose or diabetes increases cytosolic Ca^2+^ concentration and thus induces calpain activation in cultured endothelial cells and in micro/macro-vascular tissues of type-1 and type-2 diabetes [23,24,38]. To further confirm these previous findings, the present study also revealed that calpain activity was markedly increased in HUVECs stimulated with high glucose and in aortas of both STZ-induced type-1 diabetic mice and transgenic type-1 diabetic mice (OVE26 mice). The up-regulation of calpain activity in endothelial cells has been implicated in vascular inflammation and endothelial leakage in diabetes [24,26]. The present study provides additional functional evidence demonstrating that calpain activation contributes to endothelium-dependent dysfunction in diabetes. In the present study, different mouse models of diabetes including STZ-induced, genetically modified type-1 and db/db type-2 diabetes were employed. Endothelium-dependent vasodilation was impaired in all diabetic mice, confirming previous findings [9-11]. However, transgenic over-expression of calpastatin rescued the endothelium-dependent vascular relaxation in response to Ach in diabetic aortic rings. There was no alteration of vascular relaxation in response to NO donor SNP in both diabetic mice and transgenic mice with calpastatin over-expression as compared to non-diabetic and wild-type mice, respectively, suggesting that the function of smooth muscle relaxation in aortic rings of diabetic mice is preserved. It has been known that the release of prostacyclin, in particular PGI_2_, also induces endothelium-dependent relaxation in large arteries [39,40]. To exclude the involvement of those NO independent factors in calpain-mediated dysfunction of endothelium-dependent relaxation, we demonstrated that deletion of eNOS abrogated the beneficial effect of calpastatin over-expression on endothelium-dependent relaxation in diabetic aortic rings. Thus, it is highly possible that the improvement of vascular relaxation in response to Ach in diabetic calpastatin transgenic mice results from an increase in NO bioavailability. It is worthwhile to mention that a previous study has demonstrated that the predominant agonist-induced endothelium-dependent vasodilation is mediated by endothelium-derived hyperpolarizing factor (EDHF), not by NO in murine resistance vessels [41]. In the small arteries of diabetic rats, it has been shown that that NO-dependent vasorelaxation is preserved, whereas EDHF-dependent response is impaired [42]. Thus, it seems that big arteries (such as aortas) and small arteries may respond differently to diabetes in terms of vascular relaxation. Whether calpastatin over-expression could also improve EDHF-dependent response in diabetic resistance arteries needs future investigation for clarification. In addition, calpain has been shown to positively regulate eNOS activation and NO production in endothelial cells in response to VEGF [43], suggesting that the role of calpain in NO production may be dependent on distinct stimuli.

An important finding is that calpain activation mediates ROS production in vasculatures of diabetes. Intriguingly, inhibition of calpain attenuated mitochondrial superoxide generation in high glucose-stimulated endothelial cells, suggesting that calpain may play a role in mitochondrial ROS generation in diabetes. Recent findings suggest that over-generation of ROS through mitochondrial electron transport chain contributes to the diabetic vascular injury [14,18]. Uncoupling protein 2, a critical regulator of mitochondrial-derived ROS release, has been shown to attenuate the endothelial dysfunction by increasing NO bioavailability and inhibiting ROS production in diabetic mice [14,44]. In addition to the electron transport system, mitochondria also produce ROS through monoamine oxidase-dependent pathway in diabetes [13]. However, it is currently unknown how calpain activation contributes to mitochondrial ROS generation in endothelial cells. Study has shown that hyperhomocysteinemia induces the translocation of active calpain-1 from cytosol to mitochondria, leading to increased intramitochondrial oxidative stress in cultured rat heart microvascular endothelial cells [45]. In this regard, some important mitochondrial proteins have been identified as substrates of calpain-1, such as ATP5A1 [46], optic atrophy-1 [47], apoptosis-inducing factor [48], etc. Disruption of these mitochondrial proteins may induce mitochondrial dysfunction and excessive ROS generation. Nevertheless, further investigations are needed to determine the mechanisms by which calpain induces mitochondrial ROS generation in endothelial cells. Since there may be cross-talks between main ROS sources (mitochondria, NADPH oxidase, xanthine oxidase or un-coupled NOS) [49], it is very hard to determine the relative importance of individual ROS source in diabetes.

There is a general consensus that increased ROS production in the vascular wall, particularly within endothelial cells, contributes largely to the diabetic endothelial injury [1,2]. In addition to its role in promoting vascular inflammation [50,51], excessive ROS production can induce apoptotic cell death in endothelial cells [8,20]. In support of this view, we showed that inhibition of calpain prevented high glucose-induced apoptosis in endothelial cells. ROS, in particular superoxide anion reacts very rapidly and efficiently with NO to generate the extremely detrimental species peroxynitrite, reducing the bio-availability of NO production [19]. In this regard, we found that NO production was reduced in high glucose-stimulated endothelial cells and peroxynitrite formation was increased in vascular walls of diabetic mice. This is also supported by previous reports [7-9,52]. Importantly, inhibition of calpain elevated NO production in endothelial cells and attenuated the formation of peroxynitrite in aortas of diabetic mice. These data suggest that calpain activation mediates ROS generation, which in turn quenches NO, and thereby reducing its bio-availability in endothelial cells during diabetes. It is important to mention that a reduction in NO production may also result from eNOS dysfunction [3]. However, the present study found that incubation with high glucose did not change the protein levels of eNOS and phosphorylated eNOS, and the formation of eNOS dimers in endothelial cells, indicating that calpain may not directly disrupt the function of eNOS in producing NO. Indeed, previous reports have demonstrated that diabetes reduced NO bio-availability without altering eNOS protein and its dimer formation in endothelial cells [53,54]. Further evidence in support of our conclusion was that calpain cleaved eNOS protein without affecting the eNOS activity [43,55]. However, our current and these previous findings are different from a recent report which showed that calpain activation may induce the disruption of eNOS in producing NO during oxidized LDL stimulation [56]. This discrepancy may be due to different stimuli: hyperglycemia versus oxidized LDL. Therefore, we conclude that inhibition of calpain increases NO bio-availability, at least in part, by reducing ROS formation and improving eNOS function in endothelial cells under pathological conditions.

While the present study investigated the role of calpain in ROS generation in endothelial dysfunction during diabetes, it is important to mention that multiple mechanisms may be involved in calpain activation-mediated diabetic vascular complications. Calpain has been shown to target and cleave IκB, and activate NF-κB signaling, leading to inflammatory responses and apoptosis in vasculatures of diabetes [25]. Calpain may also mediate apoptosis in endothelial cells by directly targeting Bid and apoptosis inducible factor [57]. Thus, further studies will be needed to fully address the role of calpain in diabetic vascular complications.

In conclusion, we have demonstrated an important role of calpain in endothelial ROS production during hyperglycemia/diabetes, which is associated with apoptosis and a reduction in NO generation in endothelial cells. Genetic inhibition of calpain through over-expression of calpastatin reduces vascular ROS production and peroxynitrite formation, and improves endothelium-dependent relaxation in diabetic mice. Given that calpain has been implicated in vascular inflammation [24,25] and endothelial leakage [26], this study provides further evidence to support the view that calpain may serve as a potential therapeutic target for diabetic cardiovascular complications.

## Competing interest

The authors declare that they have no competing interest.

## Authors’ contributions

BC, FT and IC carried out *in vitro* studies. QZ measured calpain activity and ROS formation in aortas. RN measured mitochondrial superoxide generation in HUVECs. BC and LS assessed vascular relaxation and analyzed the data. TP designed the study. GC, WW and PS contributed to research materials. BC, GC, WW, PS and TP wrote the manuscript. All authors read and approved the final manuscript.

## Supplementary Material

Additional file 1: Figure S1 Effect of calpastatin over-expression on calpain activity. HUVECs were infected with Ad-CAST or Ad-gal, and then incubated with normal glucose (NG, 5mmol/L) or high glucose (HG, 30 mmol/L) for 48 hours. Calpain activity was determined. Data are mean ± SD from 3 different experiments. *P < 0.05 versus NG+Ad-gal and ^#^*P* < 0.05 HG+Ad-CAST. **Figure S2.** Effects of calpain inhibition on phosphorylated eNOS in HUVECs. HUVECs were incubated with normal glucose (NG, 5 mmol/L) or high glucose (HG, 30 mmol/L) in combination with calpain inhibitor-III (CI-III, 10 μmol/L) or vehicle for 48 hours. (A) A representative western blot for phosphorylated eNOS from 4 different experiments. (B) The ratio of phosphorylated eNOS to total eNOS. Data are mean ± SD, n=4. Click here for file
