# Supplementary material for: Inhibition of calpain reduces oxidative stress and attenuates endothelial dysfunction in diabetes
Source: Cardiovasc Diabetol. 2014 May 3;13:88. doi: 10.1186/1475-2840-13-88 (PMC4045988; doi:10.1186/1475-2840-13-88)
Supplement: Additional file 1: Figure S1 — Effect of calpastatin over-expression on calpain activity. HUVECs were infected with Ad-CAST or Ad-gal, and then incubated with normal glucose (NG, 5mmol/L) or high glucose (HG, 30 mmol/L) for 48 hours. Calpain activity was determined. Data are mean ± SD from 3 different experiments. *P < 0.05 versus NG+Ad-gal and #P < 0.05 HG+Ad-CAST. Figure S2. Effects of calpain inhibition on phosphorylated eNOS in HUVECs. HUVECs were incubated with normal glucose (NG, 5 mmol/L) or high glucose (HG, 30 mmol/L) in combination with calpain inhibitor-III (CI-III, 10 μmol/L) or vehicle for 48 hours. (A) A representative western blot for phosphorylated eNOS from 4 different experiments. (B) The ratio of phosphorylated eNOS to total eNOS. Data are mean ± SD, n=4. [file 1475-2840-13-88-S1.ppt]

## Slide 1
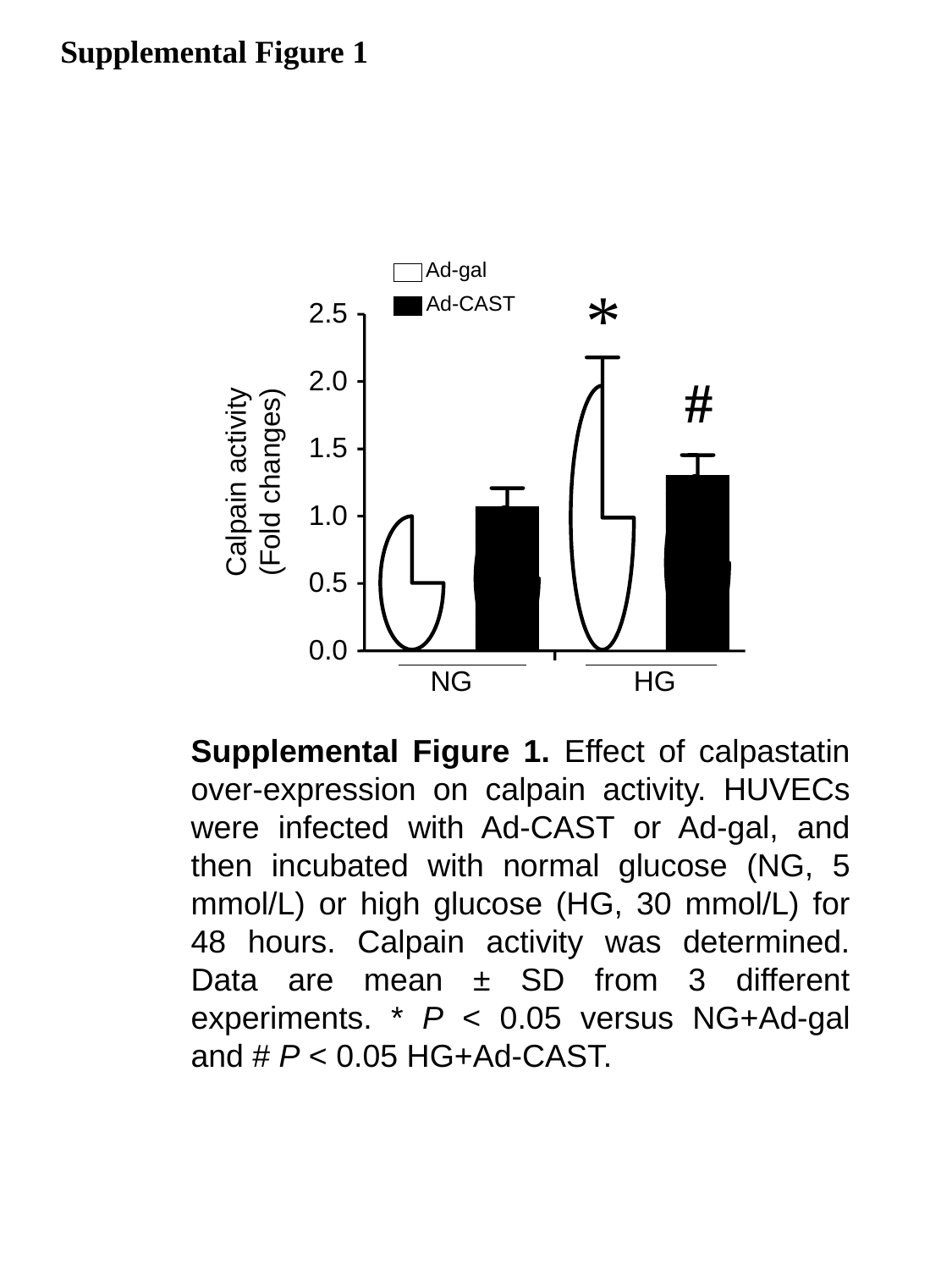

Supplemental Figure 1
Ad-gal
Ad-CAST
*
2.5
2.0
#
1.5
Calpain activity
(Fold changes)
1.0
0.5
0.0
NG
HG
Supplemental Figure 1. Effect of calpastatin over-expression on calpain activity. HUVECs were infected with Ad-CAST or Ad-gal, and then incubated with normal glucose (NG, 5 mmol/L) or high glucose (HG, 30 mmol/L) for 48 hours. Calpain activity was determined. Data are mean ± SD from 3 different experiments. * P < 0.05 versus NG+Ad-gal and # P < 0.05 HG+Ad-CAST.

## Slide 2
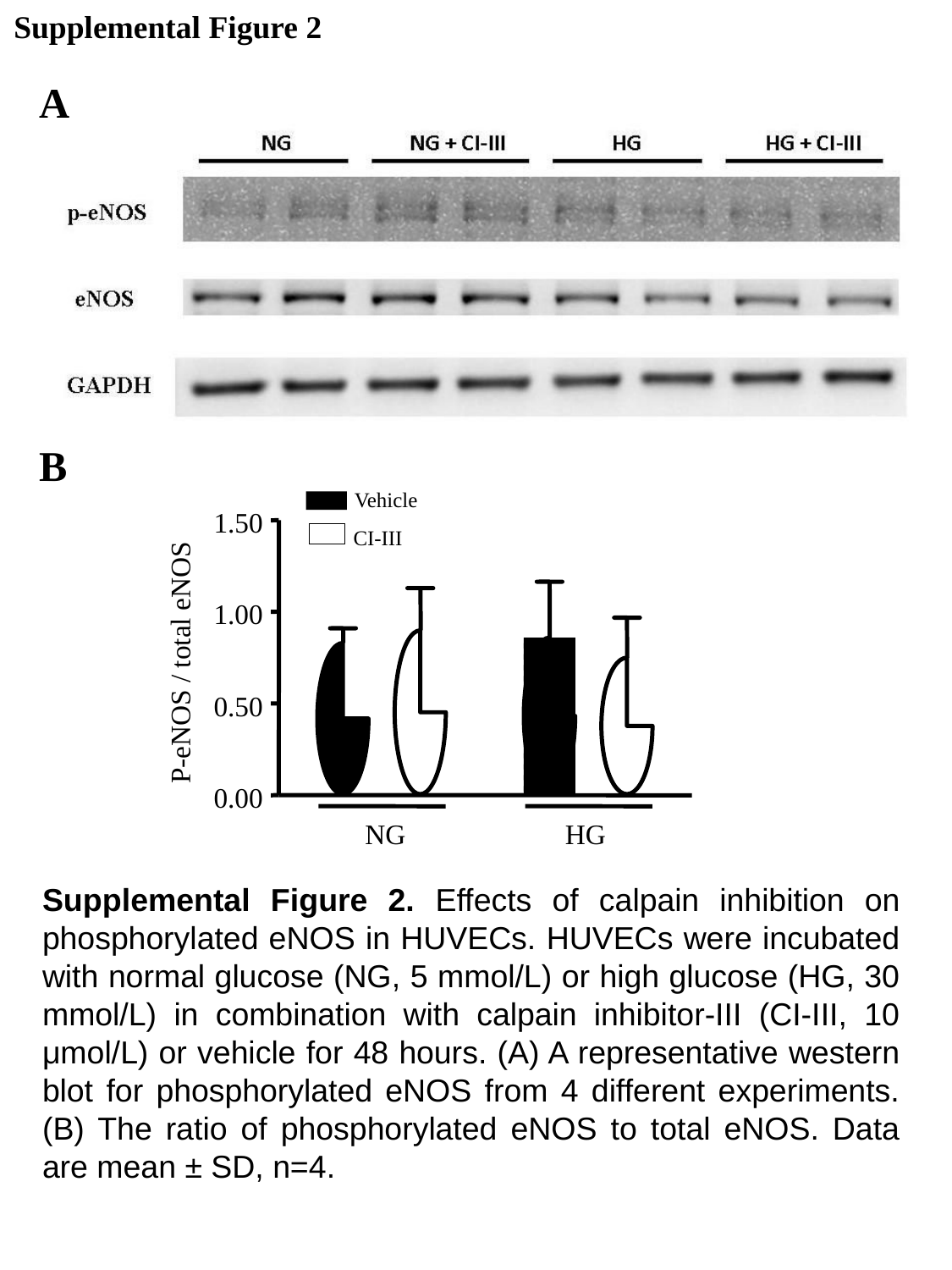

Supplemental Figure 2
A
B
Vehicle
CI-III
1.50
1.00
P-eNOS / total eNOS
0.50
0.00
NG
HG
Supplemental Figure 2. Effects of calpain inhibition on phosphorylated eNOS in HUVECs. HUVECs were incubated with normal glucose (NG, 5 mmol/L) or high glucose (HG, 30 mmol/L) in combination with calpain inhibitor-III (CI-III, 10 μmol/L) or vehicle for 48 hours. (A) A representative western blot for phosphorylated eNOS from 4 different experiments. (B) The ratio of phosphorylated eNOS to total eNOS. Data are mean ± SD, n=4.
